# Shared decision making and decisional conflict in the Management of Vestibular Schwannoma: a prospective cohort study

**DOI:** 10.1186/s40463-018-0297-4

**Published:** 2018-09-03

**Authors:** M. Elise Graham, Brian D. Westerberg, Jane Lea, Paul Hong, Simon Walling, David P. Morris, Andrea L. O. Hebb, Rochelle Galleto, Emily Papsin, Maeve Mulroy, Hannah Foggin, Manohar Bance

**Affiliations:** 1 Division of Otolaryngology – Head and Neck Surgery, Western University and London Health Sciences Centre, 5010, 800 Commissioners Road E, London, Ontario Canada; 2 Division of Otolaryngology – Head and Neck Surgery, University of British Columbia, Vancouver, BC Canada; 3 IWK Health Center and Division of Otolaryngology – Head and Neck Surgery, Dalhousie University, Halifax, NS Canada; 4 Division of Neurosurgery, Dalhousie University, Halifax, NS Canada; 5 Division of Otolaryngology, Head and Neck Surgery, Dalhousie University, Halifax, NS Canada; 6 University of Cambridge, Cambridge, UK

**Keywords:** Decisional conflict, Acoustic neuroma, Shared decision making, Vestibular schwannoma

## Abstract

**Background:**

Patients with vestibular schwannomas (VS) are faced with complex management decisions. Watchful waiting, surgical resection, and radiation are all viable options with associated risks and benefits. We sought to determine if patients with VS experience decisional conflict when deciding between surgery or non-surgical management, and factors influencing the degree of decisional conflict.

**Methods:**

A prospective cohort study in two tertiary ambulatory skull-base clinics was performed. Patients with newly diagnosed or newly growing vestibular schwannomas were recruited. Patients were given a demographic form and the decisional conflict scale (DCS), a validated measure to assess the degree of uncertainty when making medical decisions. The degree of shared decision making (SDM) experienced by the patient and physician were assessed via the SDM-Q-10 and SDM-Q-Doc questionnaires, respectively. Non-parametric statistics were used. Questionnaires and demographic information were correlated with DCS using Spearman correlation coefficient and Mann-Whitney U. Logistic regression was performed to determine factors independently associated with DCS scores.

**Results:**

Seventy-seven patients participated (55% female, aged 37–81 years); VS ranged in size from 2 mm–50 mm. Significant decisional conflict (DCS score 25 or greater) was experienced by 17 (22%) patients. Patients reported an average SDM-Q-10 score of 86, indicating highly perceived level of SDM. Physician and patient SDM scores were weakly correlated (*p* = 0.045, Spearman correlation coefficient 0.234). DCS scores were significantly negatively correlated with a decision to pursue surgery, presence of a trainee, and higher SDM-Q-10 score. DCS was higher with female gender. Using logistic regression, the SDM-Q-10 score was the only variable associated with significantly reduced DCS.

**Conclusions:**

About one fifth of patients deciding how to manage their vestibular schwannoma experienced a significant degree of decisional conflict. Involving the patients in the process through shared decision-making significantly reduced the degree of uncertainty patients experienced.

## Background

Vestibular schwannomas (VS) have an estimated incidence of 0.6–1.9 per 100,000 annually [1]. In tertiary neurotology practices, however, they can be a substantial part of the patient flow. Symptoms are variable, and it is therefore difficult to create a standardized management algorithm. Options include watchful waiting, stereotactic radiation surgery (SRS) and conventional surgical approaches. The risks and benefits of each option may make management decisions challenging for both surgeons and patients.

Watchful waiting is a commonly employed treatment approach in the modern management of vestibular schwannomas. There are no identified factors to predict an individual’s VS growth pattern at initial presentation or when the tumor first shows evidence of growth [2, 3]. Various series have shown that the majority (> 60%) of VSs do not grow if followed. If the tumor does grow, most grow slowly though some grow rapidly, with an overall estimated annual growth between 0.4 and 2.9 mm per year [4–6]. When growth does occur, it can affect patient function and influence both the options and outcomes for future management.

Microsurgical resection is another management option for patients, via the translabyrinthine, retrosigmoid or middle cranial fossa approach, with each possessing its own set of advantages and disadvantages. Surgery places surrounding nerves and arteries at potential risk. Standard neurosurgical risks also apply, such as cerebrospinal fluid leak and meningitis [7]. Stereotactic radiation surgery (SRS) as an option has its own risks. SRS can cause acute hydrocephalus from tumor swelling, albeit rarely [8], or contribute to hearing loss [9, 10]. If SRS fails, salvage surgery may be more challenging [11]. There is also the low but real risk of malignant transformation in the radiation field [12, 13].

With multiple treatment options, all with their own inherent risks and benefits, and variable natural history, any treatment decision is complex and likely to involve significant anxiety and stress. The decision between treatment options must consider the patient’s experiences, values and risk tolerance, in addition to tumor characteristics and local physician expertise. Shared decision-making (SDM) may play an important role in facilitating the management of VS. SDM is a collaborative approach that describes the process of patients working with healthcare providers to come to a consensus regarding their care. SDM has previously been shown to decrease uncertainty around management decisions and improve health-related quality of life [14, 15].

A related topic is “decisional conflict”, which defines difficulties experienced by patients in coming to a decision regarding their care. Previous data has suggested that the degree of decisional conflict experienced by patients may be influenced by the degree of shared decision-making in patient consultation [16–18], with those patients perceiving more SDM experiencing less decisional conflict. Studies in other surgical decision-making contexts have showed that patients experience significant decisional conflict when deciding between surgical and non-surgical treatment for various conditions [17, 19, 20].

The objective of this study was to determine if patients experience decisional conflict when making management decisions for their VS, and to explore which factors, if any, influence the degree of conflict experienced. Given the benign nature of the tumors and the sometimes conflicting and confusing evidence surrounding management of patients with VS, we hypothesized that these patients may experience significant levels of decisional conflict.

## Methods

### Ethical considerations

Institutional ethics board approval was obtained at both participating centers. Written informed consent was obtained from each patient and de-identified data was securely stored.

### Aim

To assess the degree of shared decision making experienced by patients when deciding how best to manage their VS.

### Participants

This study was carried out in two tertiary/quaternary academic centres. One centre (Halifax, Nova Scotia) involved a multi-disciplinary skull base clinic with fellowship trained neurotologists and neurosurgeons, whereas the second site (Vancouver, British Columbia) involved a neurotology clinic with fellowship trained neurotologists with referrals to other services being directed after the initial consultation.

All new patients with a clinical diagnosis of VS presenting to the two centers were approached. Follow-up patients with demonstrated recent VS growth on serial MRI scans were also approached, as they were faced with the need to make a new treatment decision. Patients were excluded if they declined to participate or were not fluent in English. Patients were not approached if they had previously undergone treatment of their VS.

All patients underwent a standardized clinic visit, which involved a neurotological history and examination, review of imaging, discussion of the diagnosis, and a review of possible treatment options, highlighting those felt to be most appropriate for the individual patient. Risks and benefits of each option were discussed by the attending surgeons. Following the consultation, patients who agreed to participate were referred to the research assistant who provided details of the study, obtained consent and administered the questionnaires to the patients.

### Measures

#### Demographic form

Baseline demographic information was collected, including previous surgeries, education, and income. A separate demographic form was completed by the attending surgeons responsible for the consultation including maximum diameter of VS measured on MRI, presenting symptoms, presence of a trainee, and management options presented.

#### Decisional Conflict Scale (DCS)

This 16-item Likert-like measure is a validated scale that determines patient uncertainty about a medical decision. Sample items include the following: “I am clear about what benefits matter most to me,” “I feel sure about what to choose,” and “My decision shows what is important to me.” It includes five subcategories, is context non-specific, and has been used in a variety of surgical settings [17–20]. Previous research has suggested that a score of 25 or greater is representative for significant decisional conflict [12].

#### Shared Decision Making Questionnaire-Patient Version (SDM-Q-9)

This is a validated measure with nine items on a Likert scale to assess the perception of patients regarding their involvement in clinical decision-making. Total score range from 0 (no shared decision-making) to 100 (a high degree of shared decision-making). Sample items include: “My doctor and I selected a treatment option together” and “My doctor made it clear that a decision had to be made.” The SDM-Q-9 has been shown to have high reliability [19].

#### Shared Decision Making Questionnaire-Physician Version (SDM-Q-Doc)

This is a validated scale that was developed from SDM-Q-9 to make it applicable for healthcare providers. Its format is similar to the SDM-Q-9 with overall score also ranging from 0 to 100. This scale has demonstrated high reliability [20]. Sample items include: “I wanted to know exactly from my patient how he/she wants to be involved in making the decision” and “I told my patient that there are different options for treating his/her medical condition.”

### Data analysis

Power calculation indicated that 52 patients would be required to detect a correlation coefficient of 0.38 between shared decision-making and decisional conflict. This correlation coefficient has been established in previous research examining DCS and SDM [15]. To ensure adequate sample size, accounting for attrition and incomplete data, we set the recruitment goal at 75 patients.

Data entry was completed in Microsoft Excel ™ and analysis conducted in RStudio, Version 1.0.136 (Boston, MA).

DCS was not normally distributed; therefore, non-parametric statistics were used. Descriptive statistics such as median, interquartile range and standard error (SE) are reported. Scores greater than 25 on the DCS indicate significant decisional conflict. Mann-Whitney U test and Spearman’s correlation coefficient were utilized to correlate DCS with demographic variables. Spearman’s correlation coefficient was used to compare SDM-Q-9 and SDM-Q-Doc. Logistic regression was performed to determine which factors are independently associated with decisional conflict. Statistical significance was set at *p* < 0.05.

## Results

### Patient demographics

Seventy-seven patients participated, 62 of whom (79%) were presenting to the clinics for the first time, while 15 (19%) were follow-up patients with demonstrated VS growth. One patient did not have new/follow-up patient status recorded. Fifty-eight percent of patients were recruited from Halifax, with the remainder were from Vancouver. Seventy-one percent of patients (55/78) had undergone surgery previously, with the most common surgery being a hysterectomy. Average patient age was 57.8 years old (range 37 to 81 years); 55% of patients were female and the majority (73%) were married. Patients had an average of 14 years of education (range 8 to 20 years).

### Vestibular schwannoma characteristics

The participants’ mean maximal diameter of vestibular schwannoma was 18.3 mm, with diameters ranging from 2 mm to 50 mm. The most common presenting complaint was hearing loss, noted in 92% of patients (72/78). Other presentations included vestibular dysfunction in 44%, tinnitus in 19%, and facial numbness in 17%.

### Decisional conflict

**Fig. 1:**
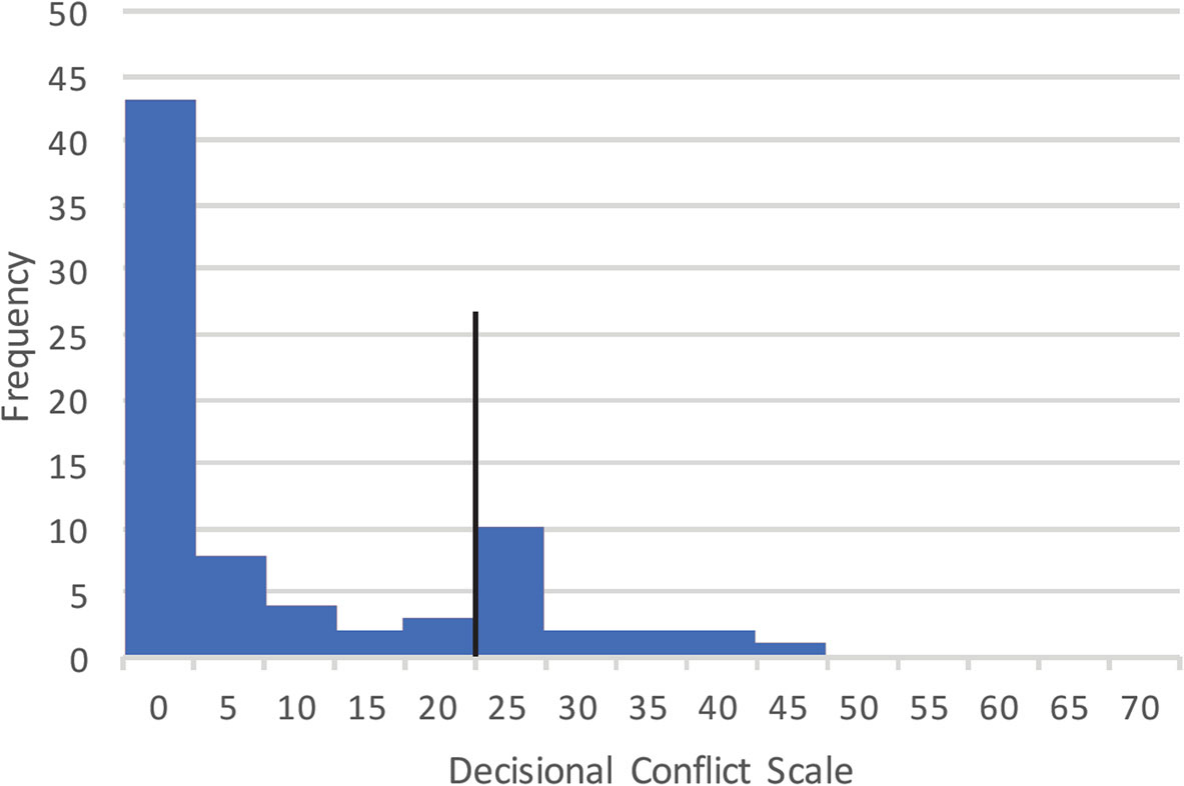
Distribution of decisional conflict amongst patients. The black line represents significant decisional conflict (25), and 22% of patients exceeded this

Median decisional conflict across all patients was 4.69. Seventeen participants (22%) had significant decisional conflict, as defined by a score of 25 or more (Fig. 1). Thirty-two patients (41%) reported that they experienced zero decisional conflict. The DCS score did not significantly differ based on previous surgery, marital status, study site, or type of visit (first time vs. follow-up). Significant differences were noted in DCS scores between participants who decided upon surgery as a treatment and those who did not (*p* = 0.034), with the surgery group experiencing lower decisional conflict. There was also a significant difference noted between the degree of decisional conflict reported by female patients and male patients (male lower), and lower DCS scores were noted in those encounters where a trainee was present (*p* = 0.035).

DCS scores were not related to whether the patient felt they wanted surgery prior to the consultation, patient age, number of previous surgeries, years of education, size of VS, or number of symptoms. DCS was also not correlated with any individual presenting symptom, including hearing loss, vestibular dysfunction, tinnitus and facial numbness (*p* > 0.05).

### Shared decision-making

**Fig. 2:**
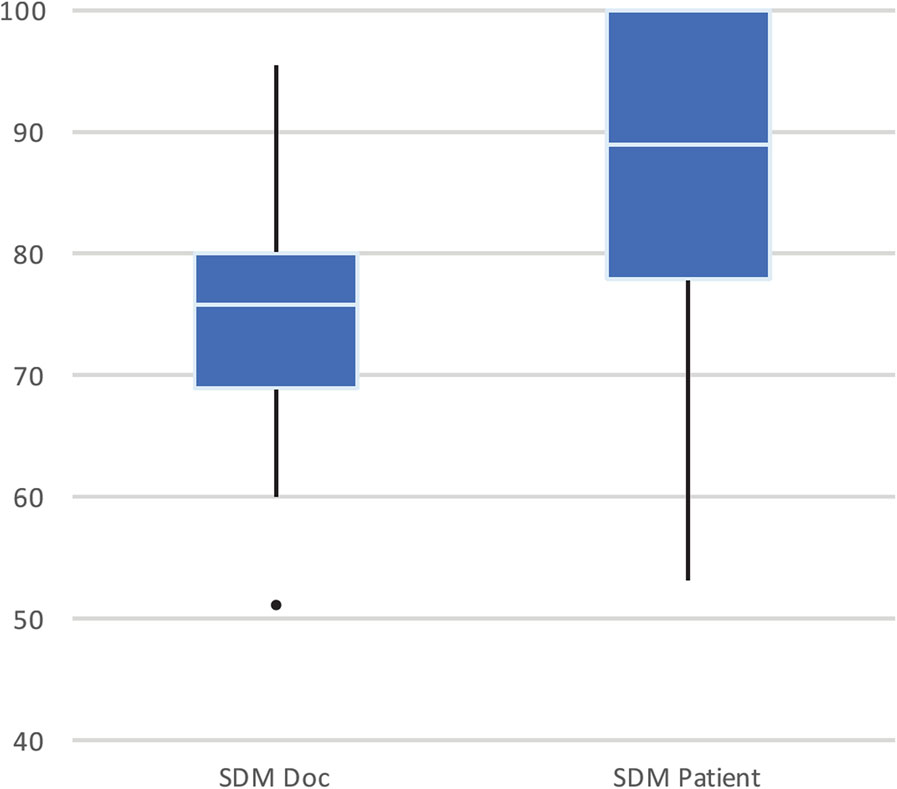
Comparison of the self-reported shared decisional conflict between patients (SDM-Q-9 (right) and physicians SDM-Q-Doc (left). The upper and lower bars represent 25th and 75th centiles, with the black dot representing an outlier

**Fig. 3:**
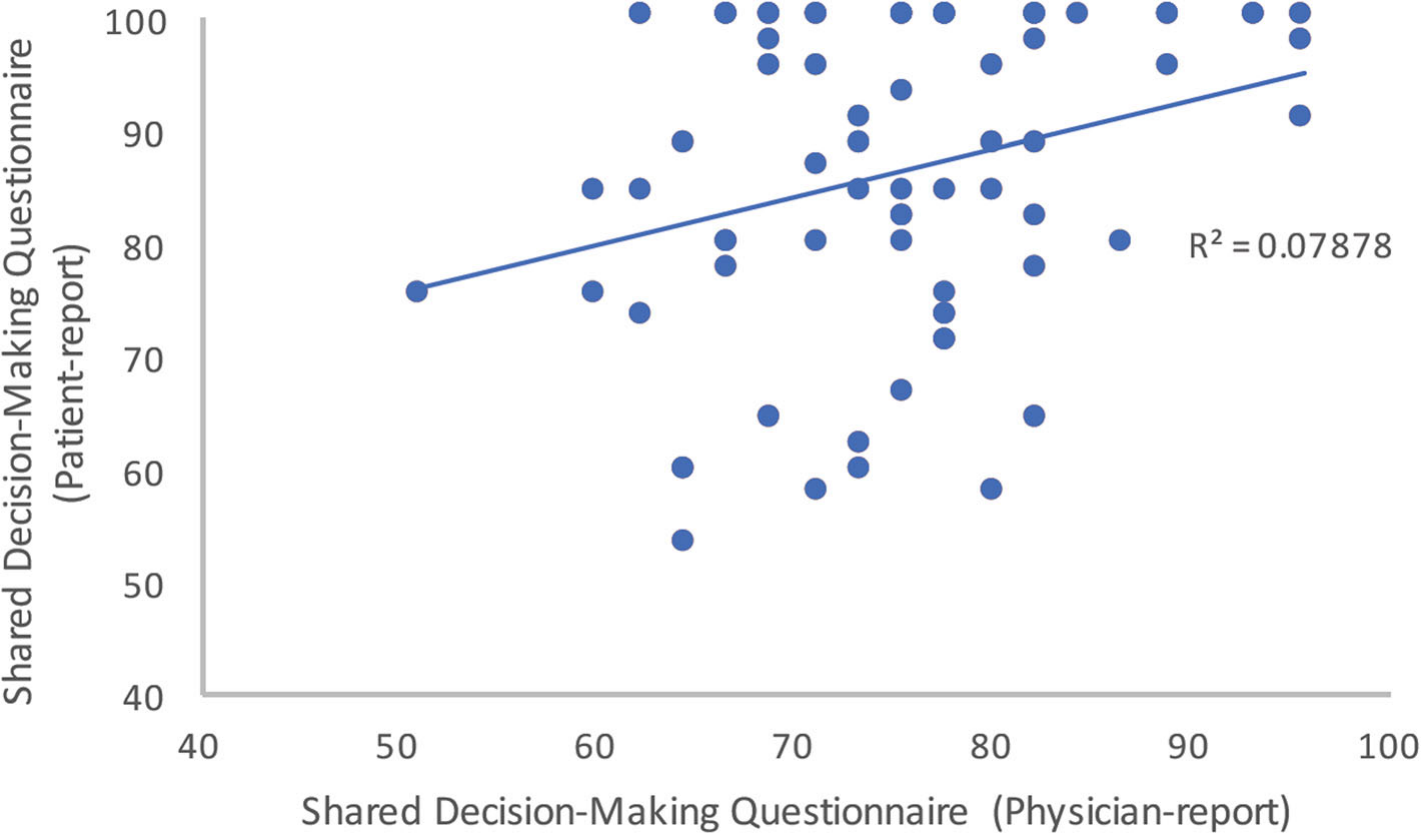
Scatter plot of the correlation between patients’ and physicians’ perceptions of shared decision making (SDM-Q-9 and SDM-Q-Doc). The *R*^2^ value is shown, correlation coefficient is 0.234

Median SDM-Q-9 scores for patients was 88.89. Median SDMQ score for physicians was 75.56 (Fig. 2). There was a significant difference between patient and physician SDMQ scores (*p* < 0.001). The mean difference in score between physician and patients was 11.29 (95% CI, 7.98–14.61). Spearman’s correlation between participant and physician shared decision-making scores was 0.234, *p* < 0.05, indicating they were weakly correlated (Fig. 3).

**Fig. 4:**
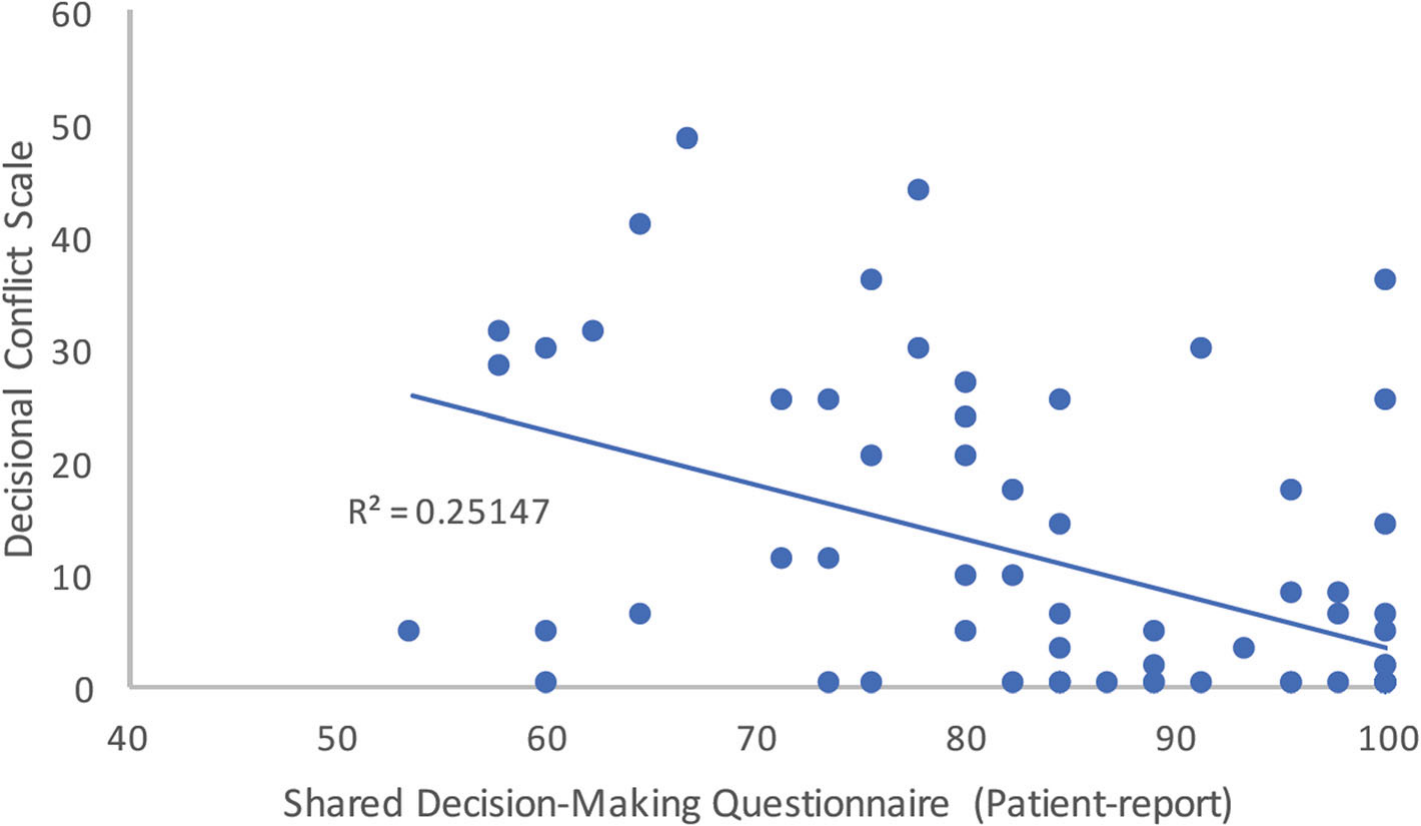
Scatter plot of the correlation between SDM-Q-9 and DCS. The *R*^2^ value is shown, correlation coefficient is − 0.539

A significantly negative correlation between SDM-Q-9 scores and DCS scores was noted (Fig. 4). SDM-Q-Doc scores and DCS scores were not correlated.

### Management decisions and decisional conflict

**Fig. 5:**
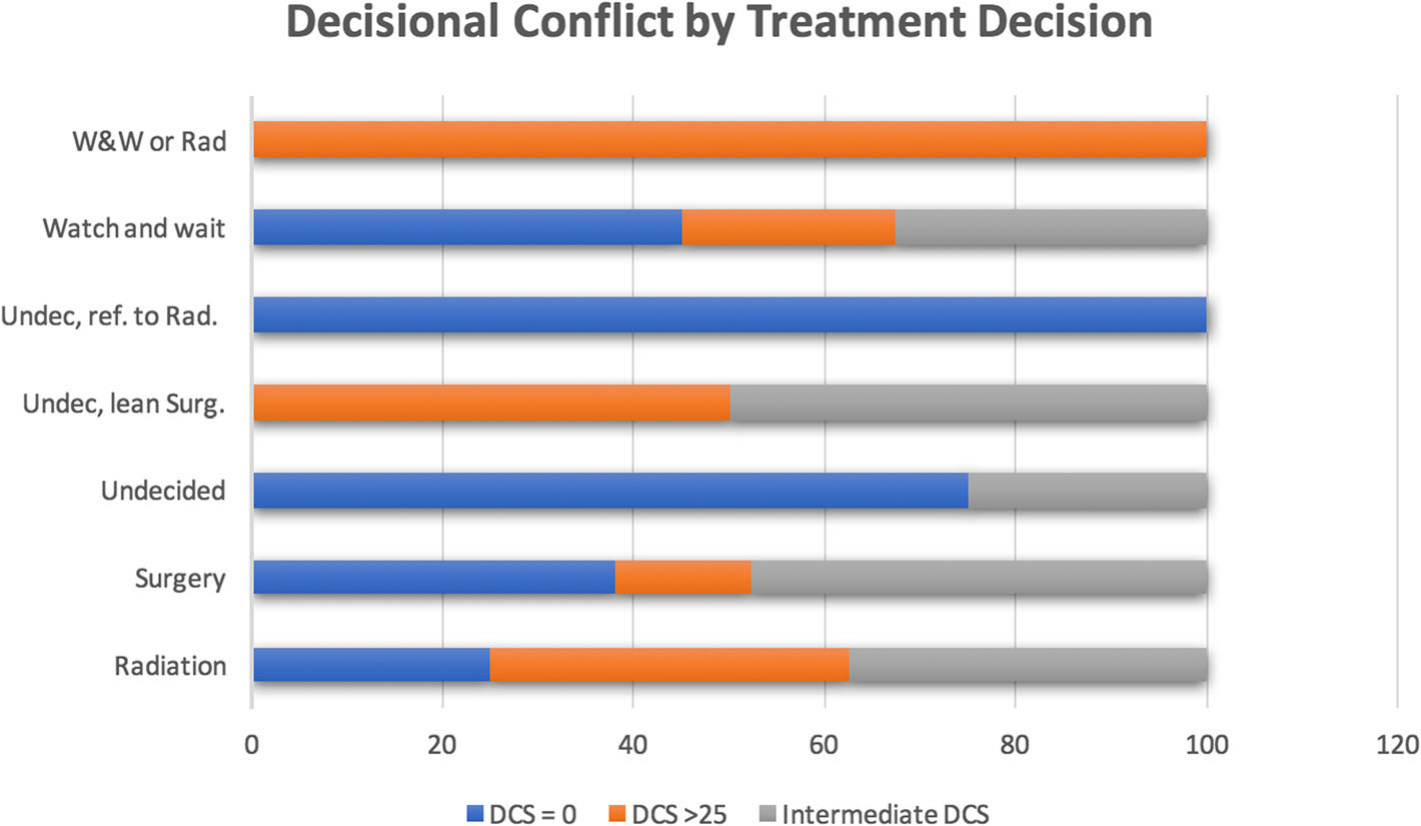
Distribution of DCS score by treatment decision. Those who remained undecided about their final treatment option (i.e. W & W or rads, undecided and leaning toward surgery) appeared to experience more decisional conflict

More than half of patients (40/78, 51%) decided on a watchful waiting approach. Twenty-one patients (27%) decided to proceed with surgery, and 8 patients (10%) decided on radiation. More than one option was usually discussed with each patient. Options discussed with patients were radiation with 88.3% of patients, surgery with 93.5%, and watchful waiting (W & W) with 85.7%. Seventy-one of 75 patients (95%) who responded to this question indicated that they knew surgery was an option prior to the consultation. Figure 5 shows the distribution of DCS by treatment decision.

### Logistic regression

Logistic regression was performed to predict the presence of significant decisional conflict (DCS > 25). The best model generated, by lowest Akaike information criterion (51.77), incorporated patient sex, age, number of previous surgeries, years of education, SDM-Q-10 score and number of options discussed with the patients to predict the presence or absence of significant decisional conflict. Of the individual variables, only the patient’s SDM-Q-10 score contributed significantly to prediction, with higher SDM-Q-10 scores predicting lower probability of significant decisional conflict.

## Discussion

### Synopsis of key findings

Given the benign nature of VS and the sometimes conflicting and confusing evidence surrounding management of VS, we hypothesized that these patients may experience significant levels of decisional conflict. The overall median decisional conflict in our study at 4.69 was well below the cut-off for significant decisional conflict. However, one of every five patients (22%) experienced significant decisional conflict (DCS > 25). Previous studies suggest that decisional conflict affects emotional wellbeing and may influence subsequent regret surrounding their management choices [21, 22].

In deciding whether to proceed with an intervention or conservative management for their VS, patients must weigh significant risks to hearing, balance, and facial nerve function. Risks of watchful waiting are that that growth may limit the ability to use SRS or that the patient may experience a general decline in health that increases the risks of surgery later. Because facial nerve and hearing outcomes from surgery are related to size of the tumor there are risks that the outcome will not be as optimal if the tumor grows prior to surgery [23]. Rates of growth are variable and not predictable. Not surprisingly, because we can only provide patients with probabilistic outcomes rather than individualized precise trajectories, we often encounter significant anxiety associated with the watchful waiting choice. This has not been well explored in previous literature.

This study suggests, as in previous studies, that patient and physician estimates of SDM were not well correlated (correlation coefficient 0.234) [15, 18, 24]. Patients overall rated a higher level of SDM than physicians did. Physicians do not seem to have an accurate sense of how involved their patients felt, although in this case the physicians underestimated their success in sharing the clinical decision. After logistic regression, shared decision making was the only factor that was significantly correlated with reduced decisional conflict. This concept clearly must become a focus of clinical consultation to improve patient experience. Physicians should examine critically their technique for presenting management options, when equivocal, to involve the patients in deciding how to manage their care.

A novel finding in our study is that the presence of trainees in the consultation appeared to decrease the degree of decisional conflict experienced by patients. The presence of a trainee may remind the consultant to use non-medicalized language, or result in additional repetition of information if the trainee discusses management options prior to the consultant entering the room. Further study may be required to elucidate the mechanism of reduced DCS.

### Comparison to other studies

Data on decisional conflict and shared decision making in patients with otolaryngology disorders is limited [15, 17, 18]. These, and studies in other surgical decision-making contexts have shown that patients experience significant decisional conflict when deciding between surgical and non-surgical treatment for various conditions [16]. For instance, almost one fifth of parents considering elective pediatric surgery for their child experienced a critical level of decisional conflict, with a DCS score greater than the predefined cutoff of 25 [15]. Decisional conflict was significantly correlated with parental perception of SDM, with patients feeling more involved in the surgical decision experiencing significantly less decisional conflict. In parents specifically considering bone anchored hearing devices for their child with aural atresia, over 40% reported experiencing significant decisional conflict [18]. This has also been shown in pediatric urology, with nearly a third of parents considering hypospadias repair for their child experiencing decisional conflict [16]. The proportion of patients with significant decisional conflict in the current study compares with data on pediatric patients undergoing elective procedures [15] but is less than adult patients considering thyroidectomy for indeterminate nodules, (34%) [17], or pediatric patients considering bone anchored hearing aids (43.5%) [18] or otoplasty (32.8%) [25]. The degree of conflict experienced by patients appears to vary considerably depending on the condition and ramifications of surgery.

Although research is increasingly showing that shared decision making is crucial in improving care, there are significant barriers to its implementation, both on the side of the healthcare provider and the patient. A systematic review by Legare et al. suggests that time constraints in a busy clinical practice remain the most frequent barrier to SDM cited by physicians [26]. Research does not presently exist showing that increasing SDM increases time of clinical encounters, however, so this may be a misconception. Physicians also may assume, at times based on socioeconomic status or other demographic factors, that patients may not desire involvement in the process of decision making [26]. Given we did not find that there was a difference in SDM or DCS based on these demographic factors, our study would strongly suggest providers not make such assumptions.

Patient identified barriers to shared decision making include inadequate provision of information to patients to allow them to make informed decisions [27]. Decision-aids are being developed in multiple areas to assist with this barrier, but improving information delivery in isolation is not enough. Other barriers identified include lack of patient knowledge that they may and should be involved in decisions regarding their care. This perception that the “doctor knows best”, and the power imbalance perceived in the doctor-patient relationship may preclude patients from participating in decisions, thinking inclusion of their values is not needed or appropriate. In vestibular schwannoma management, the neurotologist must empower the patient to be a participant in the decision-making process, giving them “permission to participate”, rather than just providing information.

### Study strengths and limitations

There are limitations to this study. The inclusion of two sites may have resulted in differing patient experiences by geographic location given differences in clinic accommodation of patients with VS. Additionally, we did not have a standardized script delivered by each provider at each visit, meaning the information presented to each patient might vary. However, this is more in keeping with what occurs in clinical practice: each patient requires directed consultation based on their presentation, and standardizing information delivery would be likely to falsely estimate the prevalence of decisional conflict in these patients. Nonetheless, the DCS scores between these two sites were not found to be significantly different in our analysis. Alternatively, the inclusion of two sites would increase the likelihood our findings could be generalizable to other skull base clinics. Most patients decided on a watch and wait approach; this may bias the degree of decisional conflict present in patients as well.

In future studies, it would be useful to follow these patients longitudinally, to determine if decisional conflict is related to the degree of decisional regret associated with decisions patients make, as seen in some previous studies [16, 22, 25].

## Conclusions

Approximately one in five patients with vestibular schwannoma experience significant decisional conflict. Increasing the degree to which the patient is involved in the decision-making process, through shared decision-making, may decrease the difficulty patients have and improve their experience in managing this potentially debilitating condition.

## Abbreviations

DCS: Decisional conflict scale
SDM: Shared decision making
SDM-Q-10: Patient shared decision making questionnaire
SDM-Q-Doc: Physician version of the SDM-Q-10
SE: Standard error
SRS: Stereotactic radiation surgery
VS: Vestibular Schwannoma
